# *Scedosporium* species in soils from various biomes in Northwestern Morocco

**DOI:** 10.1371/journal.pone.0228897

**Published:** 2020-02-24

**Authors:** Abdelmounaim Mouhajir, Wilfried Poirier, Cécile Angebault, Elkahkahi Rahal, Rachid Bouabid, Marie-Elisabeth Bougnoux, Abdessamad Kobi, Rachid Zouhair, Jean-Philippe Bouchara, Sandrine Giraud

**Affiliations:** 1 Groupe d’Etude des Interactions Hôte-Pathogène (EA 3142), UNIV Angers, UNIV Brest, Angers, France; 2 Department of Biology, Faculty of Sciences, University Moulay Ismail, Meknes, Morocco; 3 Université Paris Descartes, Service de Microbiologie, Hôpital Necker-Enfants Malades, Assistance Publique-Hôpitaux de Paris, Paris, France; 4 Department of Soil Science, Ecole National d’agriculture de Meknes, Meknes, Morocco; 5 Laboratoire en sûreté de fonctionnement qualité et organisation (EA 3858), Université d’Angers, Angers, France; 6 Centre Hospitalier Universitaire, Laboratoire de Parasitologie-Mycologie, Angers, France; University of the Basque Country, Science and Technology Faculty, SPAIN

## Abstract

*Scedosporium* species are opportunistic pathogens causing various infections, including disseminated infections in severely immunocompromised patients. Preventive measures aiming to reduce the risk of exposure to these fungi require a better knowledge on their ecology and on the sources of contamination of the patients. In this context, 99 soil samples from the Rabat-Sale-Kenitra and Fez-Meknes regions in Morocco were analyzed. Samples were inoculated on the highly selective Scedo-Select III culture medium, and seven chemical parameters of the soils were measured. *Scedosporium* species were detected in 48 of the samples, with the highest density in soils from wastewater treatment plants and landfills, followed by those from roadsides and polluted riverbanks, thus confirming the impact of human activities on their ecology. *Scedosporium apiospermum* was the most common species, followed by *S*. *boydii* and *S*. *aurantiacum*. Analysis of the chemical parameters of the soils revealed the presence of *Scedosporium* species was mainly associated with a moderate electrical conductivity, a pH range of 7.0 to 7.6, a nutrient-rich content and a moderate phosphorus amount. Thereby, these results demonstrated the relatively high occurrence of *Scedosporium* in Morocco and highlighted the impact of phosphorus content on their ecology.

## Introduction

Usually living as saprotrophic organisms, members of the ascomycete genus *Scedosporium* may cause in human a wide variety of infections affecting both the immunocompetent and immunocompromised individuals [1,2]. Various localized infections are described such as subcutaneous mycetoma, bone and joint infections, or keratitis, resulting from traumatic inoculation of some fungal elements (for example as a result of gardening, farming or traffic accidents). Disseminated infections may occur in severely immunocompromised patients like patients undergoing immunosuppressive therapy for solid organ or bone marrow transplantation [3]. Nevertheless, a particular attention has been paid to these fungi during the past decade, because of their worldwide recognition as significant pathogens in patients with cystic fibrosis (CF).

With 70000 to 100000 people affected in the world, CF is the major genetic inherited disease in Caucasian populations. Several organs are involved in this disease, but prognosis essentially depends on the severity of the lesions of the lungs. The respiratory tract of the patients is often colonized by various microorganisms, sometimes causing respiratory infections which are the major cause of morbidity and mortality in patients with CF. Bacteria are mainly responsible for these infections. Some filamentous fungi may also be encountered, including species of the genus *Scedosporium* which ranks the second among the filamentous fungi colonizing the CF lungs after *Aspergillus fumigatus*. Although usually asymptomatic, the chronic colonization of the airways by *Scedosporium* species should not be disregarded since it may lead to bronchitis and allergic bronchopulmonary mycoses, but also to severe and often fatal disseminated infections in case of immunodeficiency, particularly after lung transplantation which remains the ultimate treatment of these patients [4–7]. Therefore, all efforts should be paid to detect these fungi from respiratory secretions as early as possible, particularly at registration on the lung transplantation waiting list. Likewise, a better knowledge on the ecology of these fungi is required in order to identify the potential sources of contamination and to define preventive measures aiming to reduce the risk of contamination of the patients.

Since the first description of these fungi, a high number of papers have been published reporting their isolation from various substrates, mainly nutrient-rich substrates, such as soil and manure of livestock, poultry or cattle (for a review see [8]). Recently highly selective culture media have been developed like Sce-Sel+ or Scedo-Select III [9,10], which permitted significant progress in our knowledge on the ecology of these fungi. However, little is known about the chemical parameters that may influence *Scedoporium* abundance in the environment. *Scedosporium* species are thermotolerant fungi that have the ability to survive at very low oxygen pressure and tolerate high salt concentration and high osmotic pressure [1,11]. Kaltseis *et al*. [12] correlated *Scedosporium* abundance in the soil with increasing nitrogen concentration and decreasing pH within a pH range of 6.1–7.5. In addition, all environmental studies agreed that the ecology of this genus is strongly impacted by human activities [8]. Particularly, their common occurrence in contaminated water and highly polluted soils could be related to their ability to grow on gaseous n-alkane [13], and to use cyclic and aromatic pollutants as carbon and energy sources, like tetrahydrofuran (THF) [14], phenol [15], some polychlorinated biphenyls (PCB) [16] or rapeseed, biodiesel and diesel oils [17].

Several large scale studies dealing with the ecology of these pathogens and the distribution of the different *Scedosporium* species have been conducted, mainly in Europe, including France [18], Austria and the Netherlands [12], but also in Australia [19], Thailand [20] and South America [21,22]. They showed great variations in the relative abundance of *Scedosporium* species from one country to another. *Scedosporium apiospermum* was the most abundant species in Bangkok [23], Mexico [22] and in Austria and The Netherlands [12], whereas *S*. *dehoogii* predominated in Western France [8] and *S*. *aurantiacum* in Australia [19]. However, such studies have never been performed in Africa, and to the best of our knowledge, only sporadic isolations of *Scedosporium* sp. have been reported until now in Nigerian soils [24], and in Morocco, from the sand of two beaches in Casablanca [25] and recently from soil samples collected from Argane (*Argania spinosa*) forests in different localities of Souss-Massa region [26]. Here we report the results of an environmental study performed in the Rabat-Sale-Kenitra and Fez-Meknes regions, two of the twelve administrative divisions of Morocco, which comprise four of the ten major cities of the country. In parallel, various soil chemical parameters were evaluated to better understand the ecophysiological features of *Scedosporium* genus.

## Materials and methods

### Soil sampling

This study was conducted in the Rabat-Sale-Kenitra and Fez-Meknes regions which are located in the north of Morocco. The former, along the Atlantic ocean, presents a mild, temperate Mediterranean climate with cool winters and warm summers, while various climates can be seen in the latter: (i) a continental climate in the northern part, very hot and very dry in summer and cold and humid in winter; (ii), a cold and humid climate in the middle Atlas, very cold and very snowy in winter and temperate in summer; and (iii), a semi-arid climate in the high hills of Boulemane, where the average rainfall does not exceed 250 mm per year, very cold and snowy in winter and several days without thawing.

Ninety-nine sampling sites were selected, representing various biomes. Some samples were taken in areas exhibiting a low human activity such as the forests of Maamoura and in the national park of Ifrane (n = 10). Other sampling sites were located in the main cities of these regions and corresponded to area with high human activity: urban parks (n = 10), roadsides (n = 10), banks of two polluted rivers in Fez and Meknes (n = 10), indoor environment (mainly potted plants or garden in individual houses; n = 12), plant nurseries (corresponding to potted plants; n = 12), pedestrian city parks (n = 10) and beaches around Rabat and Kenitra (n = 13). Finally, the last samples were taken from highly polluted areas such as sewage sludge from wastewater treatment plants or the soil from a public landfill in Meknes (n = 12).

For each sampling site, plots of one square meter were defined. After clearing the surface from plant and other debris, soil samples were collected with a spatula for up to 15 cm in depth in three to five collection spots until reaching about 500 g of soil. Samples were secured in plastic bags to avoid external contamination until chemical and mycological analysis.

### Isolation of *Scedosporium* species

All soil samples were inoculated on the Scedo-Select III culture medium which was developed in our laboratory and which demonstrated its high selectivity for *Scedosporium* species in previous environmental studies [18,20]. To do this, soil samples (5 g) were mixed vigorously until homogenization (approx. 5 min) with 5 mL of sterile distilled water, and the obtained suspensions were filtered through a 100-μm-porosity nylon mesh. The filtrates were then centrifuged at 4500 rpm for 5 min. Afterwards, the pellets were resuspended into 5 mL of sterile distilled water, and 100 μL of the obtained suspensions were inoculated in triplicate on Scedo-Select III agar plates which comprises in g/L (4-hydroxybenzoate, 0.9; ammonium sulphate, 5; potassium dihydrogenophosphate, 1.25; magnesium sulphate, 0.625; ferrous sulphate, 0.01; dichloran, 0.002; benomyl, 0.008; chloramphenicol, 0.5; and agar, 20). All agar plates were incubated at 37°C for 7 days and examined daily for fungal growth.

### Species identification in the *Scedosporium* genus

The different colony types recovered on each plate were enumerated, described macroscopically and examined by light microscopy after mounting on glass slides in lactic blue stain. Fungi were identified by a mycologist on the basis of their macroscopic and microscopic features according to standard description [27].

For *Scedosporium* species identification, from 1 to 8 randomly selected colonies (mean: 3.2 colonies per positive sample) according to the number of *Scedosporium* colonies on the primary plates were subcultured for all culture-positive samples. However, isolation of *Scedosporium* colonies was not possible from 7 positive samples (*i*.*e*. 1 from urban park, 1 from indoor environment, 2 from plant nurseries and 3 from WWTP) because of the simultaneous presence on the primary plates of some extensively growing fungi (*i*.*e*. Mucorales and Aspergilli). Subcultures were performed on plates of YPDA (containing in g/L: yeast extract, 5; peptone, 10; glucose, 20; and agar, 20) pH 7.2 supplemented with chloramphenicol 0.5 g/L and cycloheximide 0.5 g/L. After checking the purity of the colonies, the isolates were stored on cryobeads at—20°C, before to be identified by matrix assisted laser desorption ionization–time of flight (MALDI-TOF) / mass spectrometry on the Andromas equipment as described by Sitterlé *et al*. [28] with slight modifications. The colony surface was scraped by swabbing and mixed with 20 μL of acid formic (80%). Aliquots (1.5 μL) of the obtained suspensions were then deposited on wells of a target plate and after drying, co-cristallized with 1 μl of matrix solution HCCA (saturated solution of α-cyano-4-hydroxycinnaminic acid, 50% acetonitrile, 2.5% trifluoroacetic acid). All isolates were analyzed in duplicate wells and the obtained spectra were compared with the reference spectral fingerprints in the Andromas database. In order to validate results from the MALDI-TOF mass spectrometry, species identification was controlled for some isolates by sequencing part of the beta-tubulin gene as described by Rougeron *et al*. [18].

### Soil chemical analysis

Soil samples were homogenized, air dried, grinded and sieved to 2 mm size. Seven chemical characteristics were determined for each soil sample (S1 Table). The pH was measured with a glass electrode using a soil-to-water ratio of 1:2.5 [29]. Stirring was maintained during pH measurement and mean values were calculated for each sample. Data were divided into classes whose limits were determined using the square root of the number of mean pH values. Theoretical amplitude of each class was determined by dividing the amplitude of the mean pH values by the number of classes.

The electrical conductivity, which reflects the amount of soluble ions in soil (total salts), was quantified in a 1:5 soil-to-water suspension [29]. Data were divided into 5 classes according to the classification proposed by Durand [30].

The total organic matter content was determined by the wet combustion according to the method of Walkley-Black [31] by treatment of an aliquot of the soil samples with concentrated K_2_Cr_2_O_7_ and H_2_SO_4_ for 18 hr. After heating, cooling at room temperature and centrifugation, the unreacted K_2_Cr_2_O_7_ in the supernatant was determined spectrophotometrically at 610 nm.

The total and active calcium carbonate content was determined using a Bernard’s calcimeter after addition of HCl 6 N [32]. Total nitrogen content was determined by the Kjeldahl method. Exchangeable potassium was determined by flame photometry after ammonium acetate extraction, and the available phosphorus was determined according to the Olsen method [29].

### Growth of *Scedosporium* on agar-based culture medium with acid pH

To check the influence of pH on the recovery of *Scedosporium* species, growth of some isolates was evaluated by measuring the diameter of the colonies every day for three weeks on agar-based YNB culture media prepared in water (pH 5.4) or with Tris/HCl buffer pH 7.4, and results were compared to those obtained on YPDA plates.

### Statistical analysis

Soil samples were grouped according to the presence or absence of *Scedosporium* species. For each chemical parameter investigated, median and first and third quartiles were determined. According to the Kolmogorov-Smirnov test and the homoscedasticity, only the pH distribution followed a normal distribution. These data were then compared using the Student’s t-test with *p* value < 0.05 for significance. For the other chemical parameter, the non-parametric Mann-Whitney test was used [33]. Multiple linear regression analysis was also performed using the SPAD^®^ software to determine the relationships between the different variables and the number of *Scedosporium* CFUs.

## Results

### Occurrence of *Scedosporium* spp. in soils from Morocco

99 soil samples from the Rabat-Salé-Kenitra and Fez-Meknes regions were analyzed by triplicate cultivation on the highly selective Scedo-Select III culture medium containing 4-hydroxybenzoate as the sole carbon and energy source. *Scedosporium* species were detected from 48 of these samples, representing a total of 1630 colonies. *Lomentospora prolificans*, previously considered as belonging to the *Scedosporium* genus, was encountered only in 5 samples, all corresponding to potted plant (3 from plant nurseries and 2 from indoor environment).

**Fig 1:**
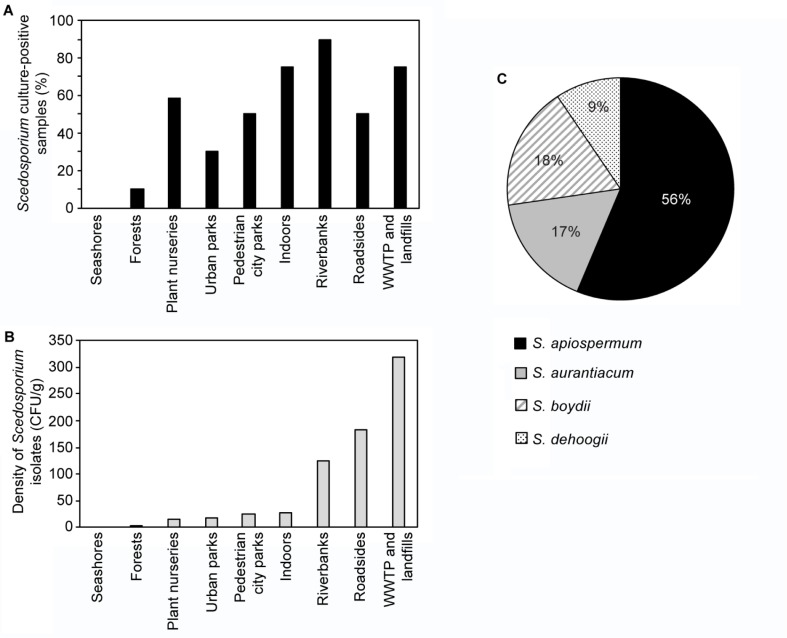
(A) Percentage of *Scedosporium* culture-positive samples (black histograms) and (B) density of *Scedosporium* isolates (grey histograms—expressed in CFU per g of soil) in soils from Rabat-Sale-Kenitra and Fez-Meknes regions, Morocco, according to the biome studied. (C) Distribution of the *Scedosporium* species among 130 isolates collected from soil samples from Rabat-Sale-Kenitra and Fez-Meknes regions, Morocco. CFU: Colony Forming Unit. WWTP: Wastewater treatment plant.

The impact of human activities on the detection of *Scedosporium* species was evidenced by determination of the number of *Scedosporium*-culture positive samples in the different biomes studied (Fig 1A and 1B). 90% and 75% of the samples from banks of polluted rivers and from wastewater treatment plants (WWTP) and landfills respectively, were culture-positive for these fungi. Likewise, the highest density of *Scedosporium* isolates was seen from the latter (mean value in positive samples: 318 CFU/g; range: 10 to > 1000). Interestingly, *Scedosporium* species were also commonly found in indoor environments (75% of the samples), but with lower densities (mean: 28 CFU/g: range: 3 to 80). About half of the samples from plant nurseries, roadsides, or pedestrian city parks revealed the presence of *Scedosporium* isolates, as well as one third of the samples from urban parks. Only roadsides yielded a high density of colonies (mean: 183 CFU/g; range: 7 to 840). In contrast, only one *Scedosporium* isolate was recovered from the protected areas corresponding to forest soils, and only seashores were *Scedosporium*-free.

Among the 1630 *Scedosporium* colonies detected, 130 colonies of the different positive samples were successfully re-isolated and preserved for precise species identification. As shown in Fig 1C, *S*. *apiospermum* represented the most common *Scedosporium* species (56% of these 130 isolates). *Scedosporium boydii* and *S*. *aurantiacum* were almost equally represented. Conversely, no *S*. *minutisporum* isolates were detected and few isolates were identified as *S*. *dehoogii* (9%). Nevertheless, it is noteworthy that the unique *Scedosporium* isolate detected from forest soils belonged to this last species.

### Influence of chemical parameters of the soils on the presence of *Scedosporium* species

**Fig 2:**
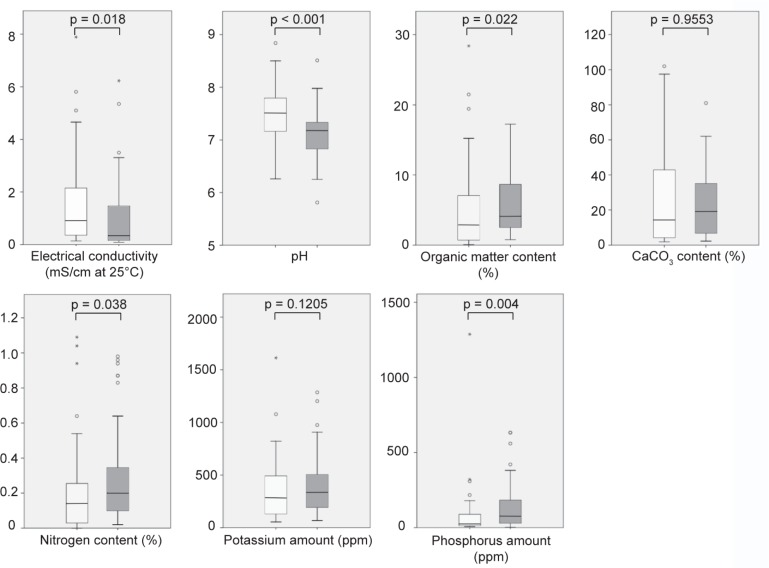
**Box plot representation of the distribution of the data obtained for the different chemical parameters studied according to the presence (gray box plot) or absence of *Scedosporium* (white box plot) species in cultures of the soil samples.** Central lines in each box indicate the median, and the lower and upper rims represent the first and third quartiles. The whiskers extend to the lowest and highest datum within 1.5 fold the interquartile range from the lower or upper quartile. Outliers are shown by circles if values are up to three times the height of the boxes or by asterisks for higher values (extreme outliers).

Seven chemical parameters were determined on the soil samples in order to explain the presence of *Scedosporium* species, including electrical conductivity (an indirect estimation of salt content), pH, total organic matter and calcium carbonate contents, and nitrogen, potassium and phosphorus amounts. Soil samples were grouped according to the presence or absence of *Scedosporium* species and data obtained for each parameter were compared between the two series of samples. As illustrated in Fig 2, distribution of the data for the electrical conductivity, the pH or the calcium carbonate content was narrower for *Scedosporium*-culture positive samples, and conversely wider for the phosphorus amount. Statistical analysis evidenced some ecophysiological features of these fungi revealing significant differences between the two groups for 5 out 7 chemical parameters: electrical conductivity, pH, organic matter content, nitrogen and phosphorus amounts.

**Fig 3:**
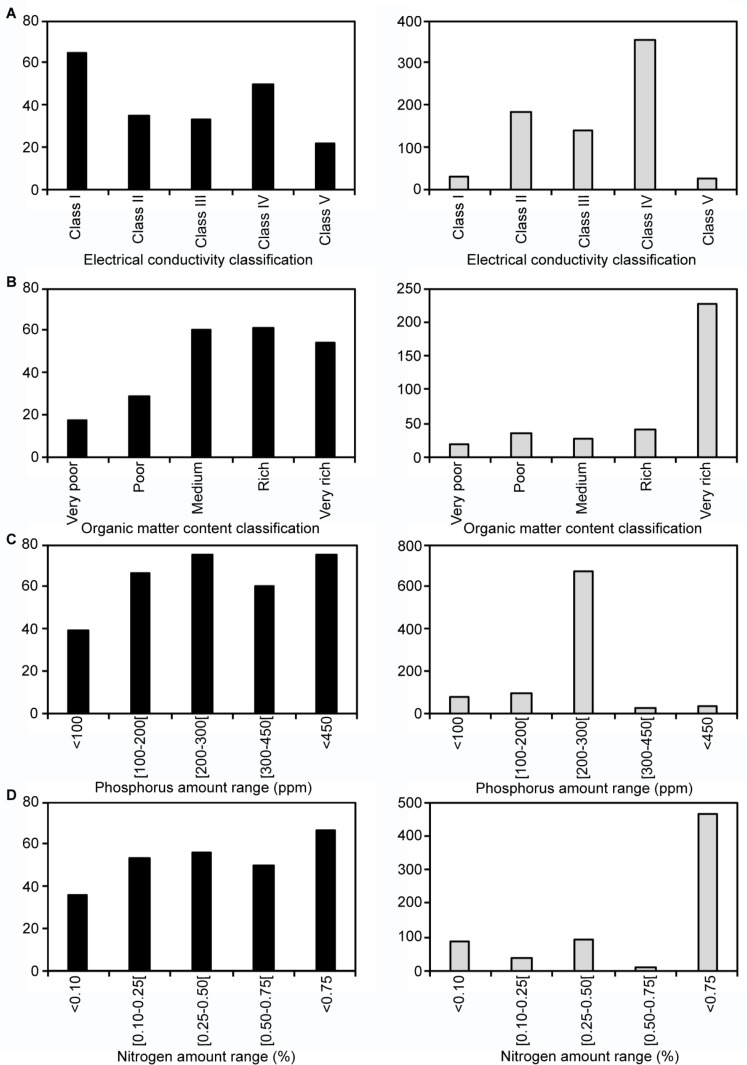
**Influence of each chemical parameter on the percentage of *Scedosporium-*culture positive samples (left panel with black columns) and on the density (CFU/g soil) of *Scedosporium* isolates (right panel with grey columns).** For each parameter, samples were distributed among five classes. (A) Based on classification by Durand (28), the electrical conductivity reflects the total ion content: class I, from 0 to 0.5 mS/cm at 25°C (no salts); class II, from 0.5 to 1 mS/cm (few salts); class III, from 1 to 2 mS/cm (salty samples); class IV, from 2 to 4 mS/cm (very salty samples); and class V, > 4 mS/cm (highly salty samples). (B) The organic matter content classes correspond to: very poor: <1%, poor: from 1% to <2%, medium: from 2% to <3%, rich: from 3% to <5% and very rich ≥5%.

For each of these parameters, the percentage of *Scedosporium* culture-positive samples (Fig 3, left panels with black histograms) and the density of *Scedosporium* isolates (Fig 3, right panels with grey histograms) were analyzed in more details, the latter one further illustrating the ecophysiological preferences. Based on the classification established by Durand JH in 1983, soil samples were divided into five classes according to the electrical conductivity (Fig 3A). *Scedosporium* isolates were found in all the classes. 50% and 65% of the samples from class I and class IV, respectively, were culture-positive for *Scedosporium* species, but with differences in density. Indeed, the mean density in the positive samples from class I, which corresponded to no salty soils, was approximately 30 CFU/g (range: 3 to 97), whereas the mean density in the positive sample from class IV corresponding to very salty soils was about tenfold higher (mean value: 355 CFU/g; range: 10 to >1000). Similar amounts of positive samples (one third) were found in the slightly salty and salty soils (classes II and III respectively), and only 22% of the samples in highly salty soils, corresponding to class V, were culture-positive for *Scedosporium* species.

The influence of the nutrient content was also well-pronounced (Fig 3B). Less than 30% of the soil samples with a very poor to poor organic matter content (< 2%) were positive for *Scedosporium* species, whereas they were from 50 to 60% among the other samples. Moreover, a higher density of *Scedosporium* isolates was observed in very rich soils (mean value: 227 CFU/g; range: 7 to >1000). Influence of the nutrient content correlated with that of the nitrogen amount (Fig 3D). Indeed, apart from those with a very low nitrogen content (< 0.10%), 50 to 70% of the samples were culture-positive for *Scedosporium*; likewise, a high density of isolates was found from nitrogen-very rich soils (mean value: 464 CFU/g; range: 30 to >1000).

As for the phosphorus content, *Scedosporium* culture-positive samples represented from 60 to 75% of soils samples, except for soils with a very low amount of phosphorus (Fig 3C). However, density of *Scedosporium* isolates was higher in soils with a phosphorus amount comprised between 200 and 300 ppm (mean value: 676 CFU/g; range: 30 to >1000).

**Fig 4:**
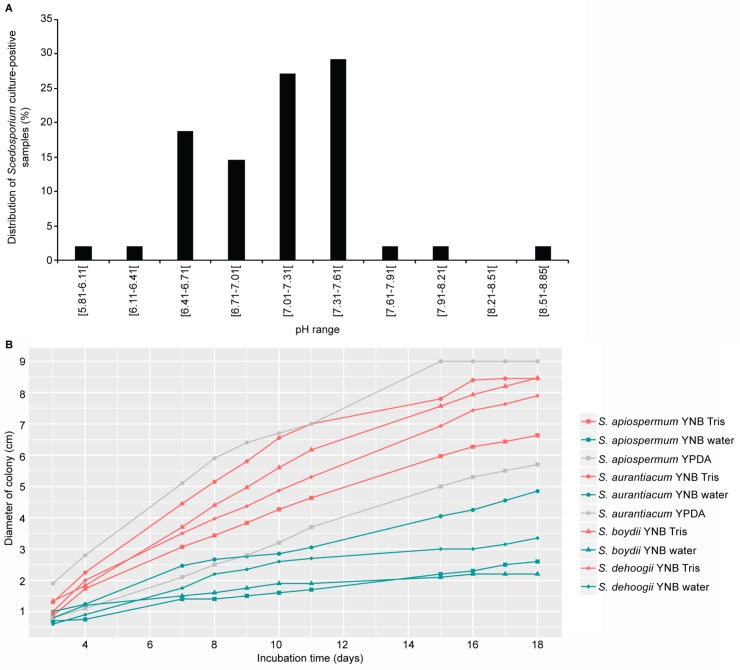
Influence of pH on the presence in soil samples (A) and on radial growth in agar-based media (B) of *Scedosporium* species. (A) Data were divided into classes whose number was determined using the square root of the number of pH values. Theoretical amplitude of each class was determined in percentage by the global addition of positive samples (B) YNB medium prepared with water exhibited an acid pH (5.4) (green curves) whereas Tris solution buffered the medium to a neutral pH value (7.4) (red curves). YPDA: Yeast extract-peptone-dextrose-agar; YNB: Yeast nitrogen base.

Considering the pH, soil samples were divided into ten classes (Fig 4A). As expected, almost all positive samples exhibited a pH within the range of 6.4 to 7.6, with a maximum number of samples showing a neutral pH comprised between 7 to 7.6. To clarify the influence of pH on the presence of *Scedosporium* species in soil samples, growth of the four major species of the genus was investigated on agar-based yeast-nitrogen base (YNB) culture media with neutral (prepared in Tris Buffer) or acid pH (usual pH with distilled water used for solubilization of the components of the culture medium). The diameter of the colonies was measured every day from day 3 to 18 following inoculation. Similar growth kinetics were observed for the four species on YNB agar plates pH 7.4 with colonies reaching between 6.5 and 8.5 cm at day 18 (Fig 4B, red curves). Compared to yeast extract-peptone-dextrose-agar (YPDA) medium (Fig 4B, grey curves), a more important or similar growth was observed on YNB at neutral pH for *S*. *apiospermum* and *S*. *aurantiacum* respectively. By contrast, although some differences were observed between species cultivated on YNB agar plates with acid pH (5.4) with a colony diameter about twice larger at day 18 for *S*. *aurantiacum* compared to *S*. *boydii* and *S*. *apiospermum*, radial growth was markedly reduced for all species with a 50 to 75% reduction in the diameter of the colony at acidic pH (Fig 4B, green curves).

**Fig 5:**
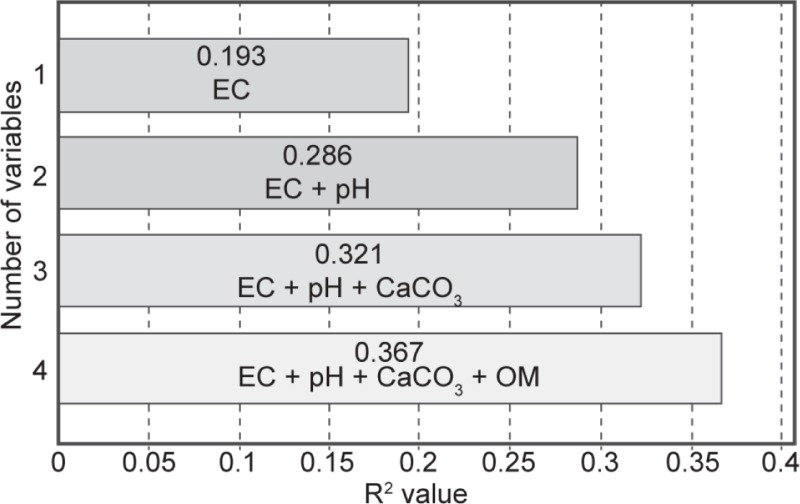
Multiple linear regression analysis of the presence of *Scedosporium* species in soil samples from Rabat-Sale-Kenitra and Fez-Meknes regions, Morocco. EC: Electrical conductivity. OM: Organic Matter.

Finally, in order to better understand the relationships between these parameters and the *Scedosporium* CFU count in the soil samples, a multiple linear regression analysis was performed according to the assumption parsimony (Fig 5). The electrical conductivity itself accounted for 19.3% of the variance of *Scedosporium* amount alone, and for 28.6% when associated with the pH. Influence of other parameters was less pronounced.

## Discussion

*Scedosporium* species are worldwide distributed filamentous fungi that have been reported from a wide variety of environments, mainly nutrient-rich substrates such as manure of livestock, poultry or cattle, guano of bats and birds, agricultural soils, and poorly aerated nutrient-rich media like muds from ditches, ponds and WWTP [8]. All studies that have been conducted so far revealed the common occurrence of these fungi in human-impacted areas [12,18,19]. Because of this, *Scedosporium* species have been proposed recently as long-term indicators of environmental pollution [34]. Indeed, *Scedosporium* species were particularly common in industrial areas, followed by urban parks and agricultural areas [12]. Likewise, a decreasing gradient in fungal density from the center of Sidney to rural areas around the city was reported by Harun *et al*. [19]. The correlation with anthropogenic impact was also evidenced in Western France where *Scedosporium* species were mainly found from WWTP, agricultural areas, city parks and playgrounds and industrial areas [18]. Conversely, these fungi never were detected from natural habitats, such as forests in Austria and sand dunes in The Netherlands [12], as well as deciduous or evergreen forests in Western France [18]. Only, two reports have been published on the isolation of *Scedosporium* sp. from the environment in Morocco. The sand of two beaches from Casablanca in Northern Morocco was sampled and one *Scedosporium* isolate was recovered from each beach [25]. Likewise, *Scedosporium* spp. represented 3.4% of fungi that were recovered in soil samples collected from Argane forests in Souss-Massa region [26]. Here we conducted the first large-scale study about the ecology of *Scedosporium* species in Africa, by the analysis of a large number of soil samples representing various biomes in Northern Morocco. 48.5% of the samples were culture-positive for *Scedosporium* species. As expected the highest fungal densities were detected from highly polluted biomes, including WWTP and landfills, roadsides and polluted riverbanks, whereas all but one of the samples from protected areas such as forests were free of these fungi. As reported in the Netherlands [12], no *Scedosporium* isolates were recovered from seashores. This result may appear conflicting with previous report of two *Scedosporium* isolates from beaches near Casablanca, the major city of the country [25], which may be attributed to the sanitary conditions of these highly visited beaches, Casablanca being the major city of the country.

Ten species are recognized today in the *Scedosporium* genus. Here, species identification was performed by MALDI-TOF/mass spectrometry according to Sitterlé *et al*. [28]. The corresponding database, however, was developed before the last taxonomic revision of the *Scedosporium* genus. The recently described species *S*. *cereisporum* is not taken into account in this database, but only two isolates are known today for this species, originating from the same sample from a WWTP in Western France [35]. In addition, *S*. *angustum* and *S*. *ellipsoideum* which are very close to *S*. *boydii*, as well as *S*. *fusoideum* and *S*. *deficiens* which are very close to *S*. *apiospermum and S*. *dehoogii* respectively, were not considered in this database [36].Taking into account this limitation, soil samples from the Rabat-Sale-Kenitra and Fez-Meknes regions revealed a high prevalence of *S*. *apiospermum*, followed by *S*. *boydii* and *S*. *aurantiacum*, with only few isolates belonging to *S*. *dehoogii*. This species distribution highly differed from that reported by Rougeron *et al*. [18] in soils from Western France where *S*. *dehoogii* was the most abundant species (39.4% of the isolates), followed by *S*. *aurantiacum* (21.6%), *S*. *boydii* (19.7%) and *S*. *apiospermum* (18.9%) being almost equally represented. Likewise, it was markedly different from that reported in the Sydney area by Harun *et al*. [19] where almost all the isolates (95.8%) belonged to *S*. *aurantiacum*. Nevertheless, weather differences between summers and winters are limited in Australia, and one may speculate that the weather in Northern Morocco is less favorable to *S*. *aurantiacum* as noticed in similar studies conducted in Europe [12,18]. However, other parameters like water capacity of soil, mineral composition and atmospheric input of chemicals may also play a role in the differences in species abundance. Interestingly, the frequency reported here for *S*. *dehoogii* is quite similar to that found for this fungus in Thailand [20]. As already noticed by Rougeron *et al*. [8], the discrepancy between the occurrence of this fungus in the environment and its paucity in human respiratory infections therefore needs to be investigated.

Besides the climate, the presence of *Scedosporium* species may also be influenced by some chemical parameters of the soils. One of the most significant parameters for *Scedosporium* presence in soils was the pH. Most of the culture-positive samples exhibited a neutral pH within a range of 7–7.6. The same pH range was also found for *Scedosporium*-positive samples in Western France [18] and Austria [12], as well as in Thailand [20,22]. The influence of pH was also illustrated by comparison of the radial growth of these fungi in agar-based media with a neutral (7.4) or acid (5.4) pH. Seashores were mainly basic or slightly basic, which may at least partly explain why these samples were free of *Scedosporium* species. Nevertheless, soils samples from forests were also within the optimal range of pH, but only one was *Scedosporium*-culture positive.

In addition, our results revealed that other soil parameters may also influence the presence of *Scedosporium*. The multiple linear regression analysis showed that the electrical conductivity was a prominent parameter to explain the presence of the target fungi. *Scedosporium* species were detected in soils whatever their electrical conductivity, but the highest densities of isolates were observed in salty soils. Our results are consistent with the ability of *Scedosporium* species to tolerate up to 5% NaCl in culture medium [9]. Electrical conductivity which is linked to the salt content may be related to the use of mineral fertilizers. Interestingly, in a previous study conducted in Western France, 11 out 12 agricultural soil samples were culture-positive for *Scedosporium*, exhibiting the highest densities of colonies among all the soil samples analyzed [18]. However, no farming lands were investigated in the present study and we do not have information about the use of fertilizers in studied plant nurseries.

Additional factors like the organic matter content and the nitrogen amount favored *Scedosporium* growth, these parameters being correlated. Indeed, the nitrogen amount was measured using the Kjeldahl method. All forms of mineral and organic nitrogen in soils had been measured except the oxidized ones; thus, the results obtained in these experiments correspond to organic nitrogen and ammonium present in soils. They indicated that a high organic matter content and high nitrogen levels are required for growth of *Scedosporium* species. This is in line with previous studies reporting a correlation between ammonium concentration and the abundance of *Scedosporium* [12].

Finally, the phosphorus amount also seemed to influence *Scedosporium* presence and growth, unlike the potassium and the calcium carbonate content. It is important to note that the Olsen method used in this study allows the determination of the assimilable phosphorus only. Whereas phosphorus is reported as the main limiting factor for biomass growth in nature [37], *Scedosporium* species were able to develop on a P-rich environment, with an optimal amount comprised between 200 and 300 ppm. This trait may at least partly explain the abundance of *Scedosporium* species in WWTP. Indeed, these environments are known to be P-rich and are considered as the main sources of phosphorus entering rivers; higher efficiency in P removal in effluents from WWTP are even one of the environmental objectives defined within the scope of European Water Framework Directive [38].

In conclusion, using a highly selective culture medium, we demonstrated a relatively high occurrence of *Scedosporium* species in the environment in Morocco and confirmed the impact of human activities on their ecology. The preferences we found for soils exhibiting high organic matter content and nitrogen level, but also a high phosphorus content, are in agreement with previous studies showing the higher density of *Scedosporium* species in human-impacted environments, especially agricultural areas because of the enrichment of farming lands by the supply nitrogen and phosphate fertilizers. Nevertheless, neither our study nor the previous ones explain *Scedosporium* preference for polluted environments.

The optimal chemical parameters of the soil for *Scedosporium* growth are: neutral pH, nutrient-rich, moderately salty to salty and a phosphorus amount within a range of 200–300 ppm. Further studies should be conducted comparing the detection rate of *Scedosporium* species with the bacterial communities in soil samples and including the determination of other chemical parameters of the soils, particularly the amount of aromatic pollutants and pesticides, as well as some physical parameters which may influence aeration and water movement like texture, structure, consistence, bulk density and pore space. Finally in addition to previous recommendations regarding potted plants which should be prohibited at home of patients with a chronic pulmonary disease, the relatively high abundance of *Scedosporium* species in human-impacted environments suggest that all situations at risk for dispersion of soil particles or fungal spores in the air should be avoided, like gardening, mowing the lawn, and of course work in polluted environments (for example wastewater treatment).

## Supporting information

S1 Table Values of the different parameters for each soil sample analysed in this study. (PDF)
