# Supplementary material for: Scedosporium species in soils from various biomes in Northwestern Morocco
Source: PLoS One. 2020 Feb 24;15(2):e0228897. doi: 10.1371/journal.pone.0228897 (PMC7039527; doi:10.1371/journal.pone.0228897)
Supplement: S1 Table — (PDF) [file pone.0228897.s001.pdf]

Table S1 : Values of the different parameters for each soil sample analysed in this study

Area type	Density of *Scedosporium* isolates (CFU/g of soil)	Parameters of the soil						
		Electrical conductivity (mS/cm at 25°C)	pH	Organic matter content (%)	Nitrogen content (%)	Potassium amount (ppm)	Phosphorus amount (ppm)	$CaCO_3$ content (%)
Forests	0	0.79	7.66	0.75	0.03	60.11	16.45	11.00
	0	4.42	6.76	2.84	0.09	164.48	16.80	2.50
	0	1.04	7.90	2.92	0.10	224.24	22.55	4.50
	0	3.37	7.45	21.47	1.04	7315.85	15.12	3.50
	0	1.35	7.29	5.16	0.18	688.52	41.65	4.00
	0	2.15	7.60	8.00	0.25	337.38	217.00	42.00
	0	0.14	7.03	13.52	0.54	241.03	6.95	2.19
	0	0.57	6.51	28.39	1.09	458.92	20.16	1.82
	3	0.33	6.62	16.68	0.59	796.60	76.45	2.56
	0	0.31	6.65	13.93	0.64	821.57	93.13	2.56
Seashores	0	0.55	8.37	0.07	0.01	56.83	10.50	70.00
	0	2.06	8.35	0.075	0.01	71.59	11.55	55.00
	0	0.56	6.38	3.59	0.14	229.00	15.05	53.00
	0	1.52	7.94	1.42	0.01	95.63	11.55	47.00
	0	4.66	7.78	0.45	0.02	151.91	18.55	10.00
	0	5.81	7.51	0.60	0.03	120.77	18.20	14.00
	0	1.27	8.50	0.30	0.02	53.55	12.60	102.00

	0	1.04	7.77	0.67	0.02	338.80	14.35	5.50
	0	1.68	8.19	0.37	0.01	71.04	10.50	2.50
	0	5.10	8.28	0.22	0.03	119.13	11.20	172.50
	0	3.99	8.31	0.15	0.02	459.06	10.50	153.00
	0	0.68	7.80	0.60	0.03	284.15	18.90	130.50
	0	3.65	6.26	1.04	0.03	154.10	7.37	41.61
**Pedestrian city parks**	0	2.71	7.11	8.34	0.26	333.00	178.75	21.90
	0	0.23	7.18	7.56	0.21	573.35	171.67	24.09
	37	0.09	7.30	1.55	0.10	66.67	9.31	2.19
	20	0.34	7.28	2.49	0.08	325.70	43.51	27.74
	0	0.17	7.24	3.32	0.08	178.69	56.30	15.33
	20	0.13	7.31	3.52	0.29	485.65	22.52	3.65
	0	0.23	7.30	3.32	0.20	767.70	52.13	23.36
	37	0.21	7.28	3.47	0.20	872.91	36.14	33.58
	13	0.21	7.32	3.68	0.15	496.35	186.96	43.80
	0	0.77	7.01	0.52	0.14	1078.26	61.16	6.21
**Urban parks**	0	0.94	8.21	2.39	0.15	592.61	22.05	55.00
	0	4.66	7.75	2.47	0.22	778.10	171.50	53.00
	0	0.51	7.79	3.44	0.19	159.02	10.50	97.50
	0	0.25	8.05	0.60	0.00	79.78	9.10	3.50
	0	0.18	6.65	3.89	0.27	191.26	27.52	43.80
	0	0.24	7.15	2.75	0.22	281.91	29.75	7.30
	13	0.14	7.19	0.88	0.06	160.11	34.19	2.92
	0	0.19	7.36	1.04	0.01	172.13	7.23	2.92
	7	0.13	7.12	5.91	0.27	672.90	110.78	2.56

	37	0.13	7.18	5.34	0.29	370.96	112.87	3.29
Plant nurseries	0	1.04	7.61	0.67	0.06	491.80	18.90	13.00
	0	1.17	7.75	2.32	0.12	90.71	39.55	9.00
	20	0.78	7.98	5.39	0.22	553.01	23.45	81.00
	0	0.36	7.41	5.61	0.17	128.96	12.25	4.00
	0	1.90	6.71	7.93	0.21	478.17	308.58	6.57
	50	1.46	6.77	11.09	0.29	741.11	631.76	6.94
	13	2.89	6.70	14.71	0.30	975.59	560.17	5.48
	7	0.19	7.60	4.20	0.06	145.36	41.28	5.11
	0	0.16	7.47	1.14	0.06	104.92	31.41	4.02
	17	0.14	7.46	0.78	0.04	98.91	14.73	3.29
	7	0.15	7.33	2.38	0.10	186.89	2.09	36.50
	3	0.15	7.42	2.02	0.10	156.83	1.39	32.12
Indoors	47	0.56	6.91	3.26	0.18	284.82	179.03	9.49
	27	0.30	7.31	2.43	0.11	193.44	75.62	8.03
	80	0.20	7.28	4.14	0.11	158.47	159.85	5.11
	27	0.26	7.17	9.53	0.36	783.88	633.15	14.60
	7	0.95	7.34	8.13	0.48	907.14	381.56	8.03
	3	0.15	7.54	2.54	0.13	472.82	8.76	8.40
	33	0.15	7.59	1.97	0.11	280.45	27.80	13.14
	20	0.14	7.59	3.47	0.11	285.55	38.23	16.43
	0	0.26	7.43	7.10	0.40	334.46	87.85	3.65
	0	0.65	7.24	8.65	0.50	438.59	131.49	4.75
	10	0.27	7.52	4.61	0.25	526.30	108.14	7.30
	0	0.38	7.18	4.25	0.35	528.43	114.12	4.38

Riverbanks	0	1.74	7.65	7.03	0.38	367.21	88.48	96.00
	90	0.36	7.42	3.52	0.20	247.60	136.36	47.45
	97	0.35	7.33	4.09	0.24	219.86	150.40	47.45
	17	1.19	6.90	12.02	0.64	446.08	305.11	25.55
	50	1.09	6.89	17.25	0.98	504.90	421.17	24.82
	53	0.23	6.91	2.07	0.04	116.94	32.94	38.33
	83	0.29	7.16	2.07	0.04	144.81	28.91	36.50
	10	0.65	6.98	7.30	0.36	306.72	73.53	43.80
	557	1.47	6.69	9.22	0.38	277.53	81.18	43.80
	170	0.80	6.99	7.10	0.33	306.72	87.57	45.99
Roadsides	0	0.87	8.03	1.35	0.07	131.15	60.55	14.00
	0	7.89	7.79	6.58	0.45	1613.20	320.60	97.00
	0	0.63	7.47	2.54	0.05	560.52	57.41	27.01
	0	0.36	7.59	2.07	0.06	531.64	94.38	23.36
	17	0.15	5.81	2.85	0.15	189.07	17.65	6.57
	7	0.21	7.06	2.28	0.03	193.99	59.21	19.35
	27	1.92	6.69	4.97	0.02	405.44	31.83	29.20
	840	0.99	6.25	6.63	0.03	374.61	38.36	24.09
	23	0.18	7.21	0.93	0.03	128.96	19.46	6.57
	0	0.34	7.02	4.61	0.08	176.50	19.32	17.52
WWTP and landfills	0	25.5	8.84	7.11	0.36	800.07	46.90	67.00
	0	2.76	6.70	15.23	0.94	327.16	165.55	19.71
	43	2.66	6.56	13.99	0.96	346.14	188.48	15.33
	1000	3.27	6.55	15.75	0.83	359.28	202.66	20.44
	30	3.19	6.56	15.49	0.87	343.22	219.20	18.98

	1000	2.79	6.53	15.28	0.87	373.88	202.66	19.71
	663	2.53	6.61	13.99	0.94	324.24	175.28	18.25
	37	6.23	8.51	2.69	0.17	6752.14	100.08	62.05
	13	5.35	7.85	5.85	0.16	6058.50	15.29	47.45
	0	3.39	7.50	19.43	0.22	41399.57	1287.14	21.90
	10	3.50	7.09	2.80	0.25	1285.69	46.15	24.09
	67	3.31	7.05	2.85	0.22	1202.45	193.91	27.74

WWTP: Wastewater treatment plants.
